# Supplementary material for: IdentPMP: identification of moonlighting proteins in plants using sequence-based learning models
Source: PeerJ. 2021 Aug 6;9:e11900. doi: 10.7717/peerj.11900 (PMC8351581; doi:10.7717/peerj.11900)
Supplement: Supplemental Information 1 — Dimension, the original dimension of feature classes. IG (Information Gain), the feature dimension after feature selection using IG. PCA (Principal Comp onent Analysis), the feature dimension after dimensionality reduction using PCA method. [file peerj-09-11900-s001.docx]

| **Feature class** | **Description** | **Dimension** | **IG** | **PCA** |
| --- | --- | --- | --- | --- |
| TPC | Tri-Peptide Composition | 8000 | 438 | 10 |
| CKSAAP | Composition of k-spaced Amino Acid Pairs | 2400 | 368 | 10 |
| CKSAAGP | Composition of k-Spaced Amino Acid Group Pairs | 2400 | 66 | 10 |
| KSCTriad | k-Spaced Conjoint Triad | 343 | 60 | 10 |
| DDE | Dipeptide Deviation from Expected Mean | 400 | 110 | 10 |
| CTDD | Distribution | 195 | 46 | 10 |
| Moran | Moran correlation | 240 | 41 | 10 |
| GTPC | Grouped Tri-Peptide Composition | 125 | 27 | 10 |
| Geary | Geary correlation | 240 | 38 | 10 |
| NMBroto | Normalized Moreau-Broto Autocorrelation | 240 | 52 | 10 |
| QSOrder | Quasi-sequence-order | 45 | 36 | 10 |
| CTDC | Composition | 40 | 32 | 10 |
| CTDT | Transition | 40 | 32 | 10 |
| PAAC | Pseudo-Amino Acid Composition | 50 | 40 | 10 |
| SOCNumber | Sequence-Order-Coupling Number | 60 | 48 | 10 |
| APAAC | Amphiphilic Pseudo-Amino Acid Composition | 25 | 20 | 10 |
