# Supplementary material for: IdentPMP: identification of moonlighting proteins in plants using sequence-based learning models
Source: PeerJ. 2021 Aug 6;9:e11900. doi: 10.7717/peerj.11900 (PMC8351581; doi:10.7717/peerj.11900)
Supplement: Supplemental Information 2 — AUPRC, area under the precision–recall curve; AUC, area under the receiver operating characteristic curve. Sen, sensitivity. Spe, specificity. MCC, Matthews correlation coefficient. F1, F1-score. For those feature classes whose information entropy of all features is less than 0.05, the dimension of feature selection is set to 70%. The maximum values in each metric are marked in bold. [file peerj-09-11900-s002.docx]

| **Feature class** | **AUPRC** | **AUC** | **Sen** | **Spe** | **MCC** | **F1** |
| --- | --- | --- | --- | --- | --- | --- |
| TPC | **0.84** | **0.86** | 0.68 | 0.85 | **0.55** | **0.70** |
| DDE | 0.73 | 0.79 | 0.62 | 0.79 | 0.42 | 0.63 |
| CKSAAP | 0.73 | 0.77 | 0.56 | 0.82 | 0.41 | 0.58 |
| KSCTriad | 0.62 | 0.67 | 0.52 | 0.66 | 0.22 | 0.48 |
| NMBroto | 0.60 | 0.67 | 0.50 | 0.74 | 0.24 | 0.52 |
| CKSAAGP | 0.60 | 0.68 | 0.33 | 0.85 | 0.27 | 0.35 |
| CTDD | 0.60 | 0.66 | 0.50 | 0.77 | 0.29 | 0.51 |
| Moran | 0.59 | 0.65 | 0.51 | 0.55 | 0.08 | 0.41 |
| GTPC | 0.58 | 0.69 | **0.72** | 0.57 | 0.28 | 0.61 |
| Geary | 0.58 | 0.66 | 0.42 | 0.66 | 0.10 | 0.39 |
| SOCNumber | 0.57 | 0.68 | 0.25 | **0.88** | 0.22 | 0.30 |
| CTDT | 0.57 | 0.67 | 0.58 | 0.68 | 0.29 | 0.55 |
| APAAC | 0.56 | 0.62 | 0.49 | 0.63 | 0.13 | 0.45 |
| PAAC | 0.54 | 0.61 | 0.41 | 0.67 | 0.10 | 0.43 |
| QSOrder | 0.50 | 0.60 | 0.46 | 0.72 | 0.18 | 0.47 |
| CTDC | 0.50 | 0.61 | 0.15 | 0.88 | 0.07 | 0.22 |
