# Supplementary material for: IdentPMP: identification of moonlighting proteins in plants using sequence-based learning models
Source: PeerJ. 2021 Aug 6;9:e11900. doi: 10.7717/peerj.11900 (PMC8351581; doi:10.7717/peerj.11900)
Supplement: Supplemental Information 5 — AUPRC, area under the precision–recall curve; AUC, area under the receiver operating characteristic curve. Sen, sensitivity. Spe, specificity. MCC, Matthews correlation coefficient. F1, F1-score. The maximum values in each evaluation metric are marked in bold. [file peerj-09-11900-s005.docx]

| **Method** | **AUPRC** | **AUC** | **Sen** | **Spe** | **MCC** | **F1** |
| --- | --- | --- | --- | --- | --- | --- |
| XGBoost | 0.43 | **0.68** | 0.46 | **0.89** | **0.37** | **0.52** |
| SVM | 0.41 | 0.66 | **0.57** | 0.71 | 0.26 | 0.49 |
| RF | 0.43 | 0.65 | 0.48 | 0.81 | 0.29 | 0.49 |
| DT | **0.50** | 0.66 | 0.57 | 0.76 | 0.32 | 0.52 |
| KNN | 0.38 | 0.56 | 0.17 | 0.86 | 0.05 | 0.22 |
